# Accelerated free-breathing 3D T1ρ cardiovascular magnetic resonance using multicoil compressed sensing

**DOI:** 10.1186/s12968-018-0507-2

**Published:** 2019-01-10

**Authors:** Srikant Kamesh Iyer, Brianna Moon, Eileen Hwuang, Yuchi Han, Michael Solomon, Harold Litt, Walter R. Witschey

**Affiliations:** 1 Department of Radiology, Perelman School of Medicine, University of Pennsylvania, Philadelphia, PA 19104 USA; 2 Department of Bioengineering, University of Pennsylvania, Philadelphia, PA 19104 USA; 3 Department of Medicine, University of Pennsylvania, Philadelphia, PA 19104 USA

**Keywords:** Parametric mapping, T1ρ, Compressed sensing, Multicoil reconstruction, Fast minimization, Endogenous contrast techniques

## Abstract

**Background:**

Endogenous contrast T1ρ cardiovascular magnetic resonance (CMR) can detect scar or infiltrative fibrosis in patients with ischemic or non-ischemic cardiomyopathy. Existing 2D T1ρ techniques have limited spatial coverage or require multiple breath-holds. The purpose of this project was to develop an accelerated, free-breathing 3D T1ρ mapping sequence with whole left ventricle coverage using a multicoil, compressed sensing (CS) reconstruction technique for rapid reconstruction of undersampled k-space data.

**Methods:**

We developed a cardiac- and respiratory-gated, free-breathing 3D T1ρ sequence and acquired data using a variable-density k-space sampling pattern (A = 3). The effect of the transient magnetization trajectory, incomplete recovery of magnetization between T1ρ-preparations (heart rate dependence), and k-space sampling pattern on T1ρ relaxation time error and edge blurring was analyzed using Bloch simulations for normal and chronically infarcted myocardium. Sequence accuracy and repeatability was evaluated using MnCl_2_ phantoms with different T1ρ relaxation times and compared to 2D measurements. We further assessed accuracy and repeatability in healthy subjects and compared these results to 2D breath-held measurements.

**Results:**

The error in T1ρ due to incomplete recovery of magnetization between T1ρ-preparations was T1ρ_healthy_ = 6.1% and T1ρ_infarct_ = 10.8% at 60 bpm and T1ρ_healthy_ = 13.2% and T1ρ_infarct_ = 19.6% at 90 bpm. At a heart rate of 60 bpm, error from the combined effects of readout-dependent magnetization transients, k-space undersampling and reordering was T1ρ_healthy_ = 12.6% and T1ρ_infarct_ = 5.8%. CS reconstructions had improved edge sharpness (blur metric = 0.15) compared to inverse Fourier transform reconstructions (blur metric = 0.48). There was strong agreement between the mean T1ρ estimated from the 2D and accelerated 3D data (*R*^2^ = 0.99; *P* < 0.05) acquired on the MnCl_2_ phantoms. The mean R1ρ estimated from the accelerated 3D sequence was highly correlated with MnCl_2_ concentration (R^2^ = 0.99; *P* < 0.05). 3D T1ρ acquisitions were successful in all human subjects. There was no significant bias between undersampled 3D T1ρ and breath-held 2D T1ρ (mean bias = 0.87) and the measurements had good repeatability (COV_2D_ = 6.4% and COV_3D_ = 7.1%).

**Conclusions:**

This is the first report of an accelerated, free-breathing 3D T1ρ mapping of the left ventricle. This technique may improve non-contrast myocardial tissue characterization in patients with heart disease in a scan time appropriate for patients.

**Electronic supplementary material:**

The online version of this article (10.1186/s12968-018-0507-2) contains supplementary material, which is available to authorized users.

## Background

In cardiomyopathy patients, myocardial fibrosis is prognostic for greater risk of sudden cardiac death, development of heart failure, and greater rates of rehospitalization [1]. Patients with suspected fibrosis are often referred for gadolinium enhanced cardiovascular magnetic resonance (CMR) due to the late accumulation and slow release of gadolinium-based contrast agents (GBCAs) from the fibrotic tissue [2, 3]. For many patients with advanced renal disease, GBCAs are contraindicated due to poor filtration of the contrast agent and retention of gadolinium in the body [4]. To address this problem, there has been a recent resurgence of endogenous contrast imaging and relaxation time mapping methods, such as T1 [5, 6] and T1ρ CMR, for non-GBCA assessment of myocardial fibrosis [7–9].

Cardiac T1ρ CMR, also called T1 in the rotating frame, uses a moderate amplitude radiofrequency pulse (i.e. spin locking pulse), as the contrast-generation mechanism [10, 11]. The spin locking pulse has been shown in preclinical validation experiments and in patients to suppress background sources of transverse relaxation, and improve the dynamic range between water ^1^H relaxation in fibrotic and normal myocardial tissues [12–15]. However, existing 2D cardiac T1ρ methods have limited spatial coverage and resolution, limiting the detection of myocardial fibrosis [10, 11]. Multiple long breath-holds are required for whole heart coverage. This can lead to misalignment of images, repeat scans and an increase in patient discomfort. A 3D method was implemented for large animals [11], but is not appropriate for patients due to long scan times and use of ventilator gating in anesthetized animals.

Compressed sensing (CS) [16, 17] can be used to reconstruct high quality images from undersampled k-space data by leveraging signal sparsity in a relevant image transform domain. CS uses iterative techniques to minimize the reconstruction cost functional, balancing data fidelity and regularization. Some CS T1ρ techniques have been developed for applications such as imaging the brain [18], knee [19, 20] and spine [18]. Depending on the application, different sparsifying transforms and reconstruction formulations were developed to reconstruct artifact free images from the undersampled data. Although these techniques have shown high quality reconstruction for their specific applications, their reconstruction speed is relatively slow. These reconstruction formulations do not use rapid minimization techniques such as Split Bregman [17] or Augmented Lagrangian [21]), which have been shown to accelerate the convergence of CS reconstructions. These alternating direction method of multipliers (ADMM)-based techniques use variable substitution to separate the sparsity and data fidelity terms and this decoupling allows for accelerated convergence.

A recent a study [22] compared several sparsity and low rank models and found that spatial-temporal finite difference reconstructions performed well at reconstructing high quality images from undersampled knee cartilage data. To accelerate convergence, approaches based on the application of fast iterative shrinkage-thresholding algorithm (FISTA) [23] and its variants were developed. The reconstruction speed of the sparse and low rank methods might be further improved by using Split Bregman or Augmented Lagrangian implementations, though no such methods are currently available for accelerated 3D T1ρ CMR. Additionally, the performance of these CS techniques has not been prospectively assessed for 3D cardiac T1ρ mapping or in other organs. There are no methods available for accelerated 3D myocardial T1ρ mapping.

We aim to develop an accelerated free-breathing whole heart 3D T1ρ mapping technique using CS. We use a modified k-space ordering technique to reduce cardiac and respiratory motion degradation, and develop a rapid reconstruction formulation based on a novel combination of Split Bregman -based variable substitution and Fourier minimization for multi-coil CS. We analyze the sources of T1ρ error using Bloch simulations and phantoms. Finally, we show feasibility, accuracy and repeatability of the technique for left ventricle T1ρ mapping.

## Methods

### Bloch simulations

We performed Bloch simulations to measure the accuracy of 3D T1ρ maps for numerical phantoms with T1ρ relaxation times that approximated normal (T1ρ = 60 ms and T1 = 1000 ms) and infarcted (T1ρ = 120 ms and T1 = 1200 ms) myocardium [11, 24], and assessed the effect of variations of the measured k-space signal and data undersampling on image blurring. We implemented a 3D T1ρ-prepared balanced steady-state free-precession (bSSFP) sequence consisting of a T1ρ preparation period, a bSSFP magnetization signal transient stabilization period and bSSFP readout [10]. The T1ρ preparation consisted of a composite radiofrequency (RF) pulse utilizing a spin echo, spin lock (SL) (90_x_-SL_y_-180_y_-SL_-y_-90_-x_) [25]. Prior to spatial encoding, a second magnetization preparation was performed using a bSSFP flip angle ramp [10]. Spatial encoding was performed using a bSSFP readout. The simulation parameters were flip angle = 70°, number of segments/readout echoes per heartbeat (N_seg_) = 48, number of shots/heartbeats (N_shot_) = 24, heart rate = 60 bpm, spin lock B_1_ = 500 Hz, TE/TR = 1.4/2.8 ms, and the time between T1ρ preparation was 2 s. Bloch simulations were performed by solving the piecewise time-independent matrix solution to the Bloch equations [10].

### Undersampling pattern and k-space sample ordering

Undersampled k-space data (A = 3) was acquired using a variable density sampling pattern, fully sampled along *k*_*x*_, while directions *k*_*y*_, *k*_*z*_ were undersampled using a bell shaped polynomial variable density distribution [26] given by

1 $$ P\left({\mathrm{k}}_y,{k}_z\right)={\left(1-r\left({k}_y,{k}_z\right)\right)}^p $$

where *r* is the normalized distance from the k-space center. Samples close to the center had high sampling probability while samples further away had low sampling probability. The polynomial order (*p*) controlled the k-space sampling density.

A k-space ordering technique was devised to minimize motion inconsistencies between heart beats due to respiratory motion. The total number of sampled k-space locations were divided evenly into the total number of shots (N_shot_), where each shot had N_seg_ sampled locations. Points that were closest to the center of *k*_*y*_-*k*_*z*_ were sampled in the earliest shot. Within a shot, a linear (column wise) sorting order was used.

### Image blur and T1ρ accuracy

We determined the combined effects of the sequence magnetization response function and data undersampling on image quality (blur) and T1ρ relaxation time accuracy using a cylindrical phantom simulation. Image quality was analyzed using the blur metric [27]. The blur metric is normalized to the range [0,1], and a large blur metric corresponds to more blurring of the edges in the image. The relaxation time estimated from the reconstructed images was compared with the known T1ρ value used for the Bloch simulations and the error was calculated.

### Phantom experiments

Experiments were performed on phantoms to compare the accuracy and repeatability of the accelerated 3D sequence to the 2D T1ρ sequence [28]. Five cylindrical phantoms were prepared in 15-ml conical tubes of distilled water and 0.01, 0.03, 0.05, 0.07 and 0.09 mM MnCl_2_ and the outer compartment contained tap water. For both 2D and 3D T1ρ acquisitions, data were acquired using 7 spin lock times (TSLs) (TSL = 2, 10, 18, 26, 34, 42, 50 ms). The sequence was triggered with a simulated heart rate of 60 bpm and data were acquired with 100% navigator efficiency. This corresponds to a scan time of 0.8 min/TSL for the 3D acquisitions and 14 s for the 2D acquisition. Other scan parameters for the phantom experiments were the same as detailed below in the human subject sections. Data acquisition was repeated three times to test for repeatability.

### Healthy human studies

**Fig. 1:**
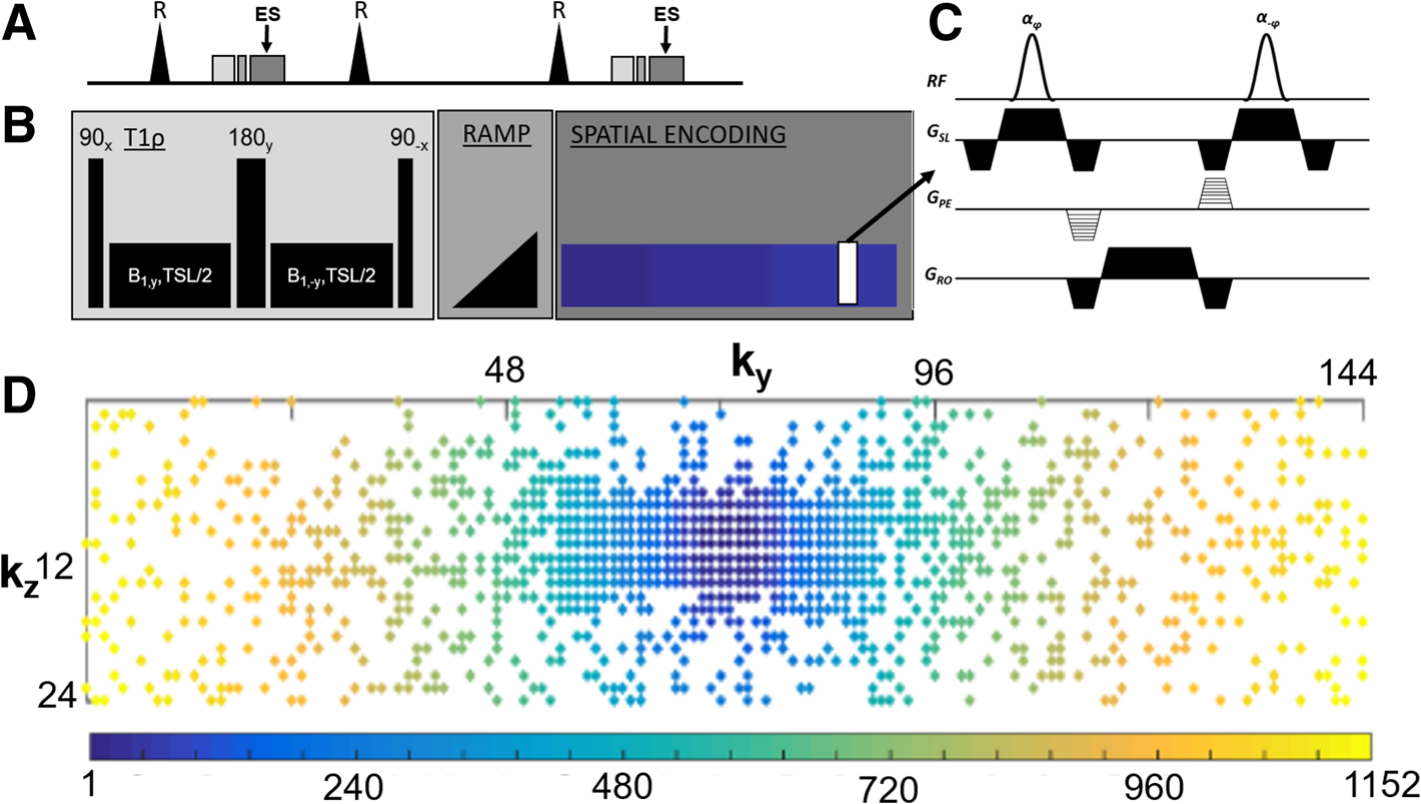
Pulse sequence for electrocardiogram (ECG) and navigator gated 3D T1ρ and accelerated k-space sampling order schema. **a** Image gating is synchronized with end-systole to achieve maximum myocardial wall thickness during this cardiac no motion period. Three heartbeats are shown. Sampling occurs every other heartbeat to allow for longitudinal relaxation of magnetization. **b** T1ρ and magnetization stabilization (ramp) followed by spatial encoding of a k-space subset is performed during the end-systolic time. **c** Enhanced view of the balanced steady-state free-precession kernel used during ramp and spatial encoding. **d** K-space sample ordering is shown. In this example, a subset of kspace (i.e. 48 k-space phase encoding lines) are acquired per heartbeat. K-space sample ordering is performed to assure that center spatial encoding frequencies are collected in the same heartbeat as much as possible. The color bar indicates the k-space ordering index

The study was approved by the institutional review board (IRB) of the University of Pennsylvania and informed consent was acquired from the subjects prior to data acquisition. Eight prospectively undersampled (A = 3) 3D T1ρ datasets were acquired from six healthy subjects on a 1.5 T scanner (Avanto; Siemens Healthineers, Erlangen, Germany) equipped with an 18 channel anterior body and posterior spine RF coil arrays using a T1ρ-prepared bSSFP sequence and a spin echo, SL T1ρ pulse cluster (90_x_-SL_y_-180_y_-SL_-y_-90_-x_) and B_1_ = 500 Hz. T1rho weighted images were acquired at 12 TSL’s (TSL = 2, 5, 8, 10, 15, 20, 25, 30, 35, 40, 45, 50 ms). The images were acquired at a 1.9 × 1.9 × 6 mm^3^ spatial resolution using a (192 × 144 × 24) acquisition matrix. Other imaging parameters were TR/TE = 2.8/1.53 ms, BW = 898 Hz/pixel and flip angle = 70°, and asymmetric echo. Data were acquired using electrocardiogram (ECG)-gating to end-systole and respiratory navigator-gating to end-expiration. ECG gating and navigator triggering was performed to reduce the effects of cardiac and respiratory motion. The end-systolic phase was determined by examining the cardiac motion from a short-axis cine scan and the beginning of the spatial encoding was adjusted such that T1ρ-weighted images were acquired during end-systole, which corresponds to maximum wall thickness. Imaging was performed every other heartbeat. For a 60 bpm heart rate and 50% navigator efficiency, the scan time for acquiring one T1ρ-weighted 3D dataset was ~ 1.6 min. The navigator efficiency varied between patients and also between the different TSL weighted scans within a patient. The mean navigator efficiency for acquired datasets was (54.4% ± 12) and the total scan time for the human scans was ~ 18 min. A 2D T1ρ scan was performed on a mid-ventricular slice for each human subject. The 2D T1ρ images were acquired with a 1.9 × 1.9 mm resolution and 8 mm slice thickness. T1ρ weighted images were acquired at 7 TSL’s (TSL = 2, 10, 18, 26, 34, 42, 50 ms). Data acquisition as performed every other heart beat. A total of 14 heart beats were required for the 2D breath-held acquisitions. The scan for the 2D acquisition varied between subjects depending on their heart rate. For a nominal heart-rate of 60 bpm, the scan time for the 2D breath-held scan was 14 s. The 3D acquisition was repeated twice on two subjects to test for repeatability. The pulse sequence for ECG and navigator gated 3D T1ρ and the accelerated k-space sampling order schema is shown in Fig. 1.

### CMR image reconstruction

Undersampled k-space data was reconstructed using a multicoil CS formulation. The unconstrained problem is given by:

2 $$ C=\frac{\mu }{2}\sum \limits_{i=1}^{Nc}\left\Vert {EC}_im-{k}_i\right.\left\Vert {}_2^2\right.+\lambda {\left|{\nabla}_{xyz}m\right|}_1 $$

Here *E* is the 3D encoding matrix, that includes the sampling pattern and the Fourier operator, *C* is the coil sensitivity map, *N*_*c*_ the total number of coils, *k* is the measured k-space data and *∇*_*xyz*_ is the 3D spatial gradient operator used to apply the 3D total variation (TV) constraint [17]. The cost functional is rapidly minimized using the application of the Split Bregman based variable substitution technique [17] by applying the following two variable substitutions, *S* = *∇*_*xyz*_*m* and *P*_*i*_ = *C*_*i*_*m*. Enforcing the two variable substitutions using Split Bregman, eq. (2) is rewritten as

3 $$ {\displaystyle \begin{array}{c}\min {P}_i,m,S,T,{Q}_i\kern0.5em \frac{\mu }{2}\sum \limits_{i=1}^{Nc}\kern0.5em \parallel {EP}_i-{k}_i\operatorname{}\parallel {}_2^2\operatorname{}+\frac{\alpha }{2}\sum \limits_{i=1}^{Nc}\kern0.5em \parallel {P}_i-{C}_im-{Q}_i\operatorname{}\parallel {}_2^2\operatorname{}\\ {}+\\ {}\lambda {\left|S\right|}_1+\frac{\beta }{2}\kern0.5em \parallel S-{\nabla}_{xyz}m-T\operatorname{}\parallel {}_2^2\operatorname{}\end{array}} $$

Here *S* and *P*_*i*_ are the surrogate variables and *Q*_*i*_ and *T* come from optimizing the Bregman distance [17]. The variables *P*_*i*_ and *m* are present only in L_2_ norm terms and are decoupled from the L_1_ norm term.

4 $$ \min {P}_i\kern0.5em \frac{\mu }{2}\sum \limits_{i=1}^{Nc}\kern0.5em \left\Vert {EP}_i-{k}_i\right.\left\Vert {}_2^2\right.+\frac{\alpha }{2}\sum \limits_{i=1}^{Nc}\left\Vert {P}_i-{C}_im-{Q}_i\right.\left\Vert {}_2^2\right. $$

5 $$ \min m\kern0.5em \frac{\beta }{2}\parallel S-{\nabla}_{xyz}m-T\operatorname{}\parallel {}_2^2\operatorname{}+\frac{\alpha }{2}\sum \limits_{i=1}^{Nc}\kern0.5em \parallel {P}_i-{C}_im-{Q}_i\operatorname{}\parallel {}_2^2\operatorname{} $$

The terms *Q*_*i*_ and *T* are minimized using a simple linear update step [17]. The iterations were terminated when a convergence criterion was met or the maximum set of iterations was reached. Pseudocode of the reconstruction algorithm is provided as Additional file 1: Figure S1.

The reconstruction method was implemented in MATLAB™ (Mathworks, Natick Massachusetts, USA) and tested on a computer with an intel Xeon E5–2603 CPU with a processor frequency of 1.8 GHz, 8 cores and a total memory of 64GB. The weights for the cost functional were chosen based on a test dataset. The weights were chosen as *μ* = 0.6, *λ* = 0.9, *α* = 0.1 and *β* = 0.1. The set of weights chosen allowed for the rapid convergence of the cost functional. The maximum number of iterations was set to 100 and the iteration was terminated when a set tolerance level was reached.

### CMR parametric mapping

Motion correction using diffeomorphic registration [29] was performed prior to quantification. This has been shown to improve the quality of T1ρ fit [10]. T1ρ maps were generated from the images by fitting a two parameter signal model [30] given by

7 $$ S={S}_0{e}^{\left(\frac{- TSL}{T1\rho}\right)} $$

where S_0_ is the initial magnetization and TSL is the spin-lock pulse duration.

### Statistical analysis

All descriptive quantities are reported as mean and standard deviation (mean ± SD). Comparisons of 2D and 3D T1ρ phantom scans were performed using linear regression, intra-class correlation coefficient (ICC), coefficient of variation (COV) and Bland-Altman plots. The mean R1ρ for the five phantoms was compared with the concentration of MnCl_2_ using linear regression and Pearson’s r. For the in vivo cardiac acquisitions, myocardial T1ρ maps were divided into six regions and the mean, SD and COV were computed.

## Results

### Bloch simulations and k-space ordering

**Fig. 2:**
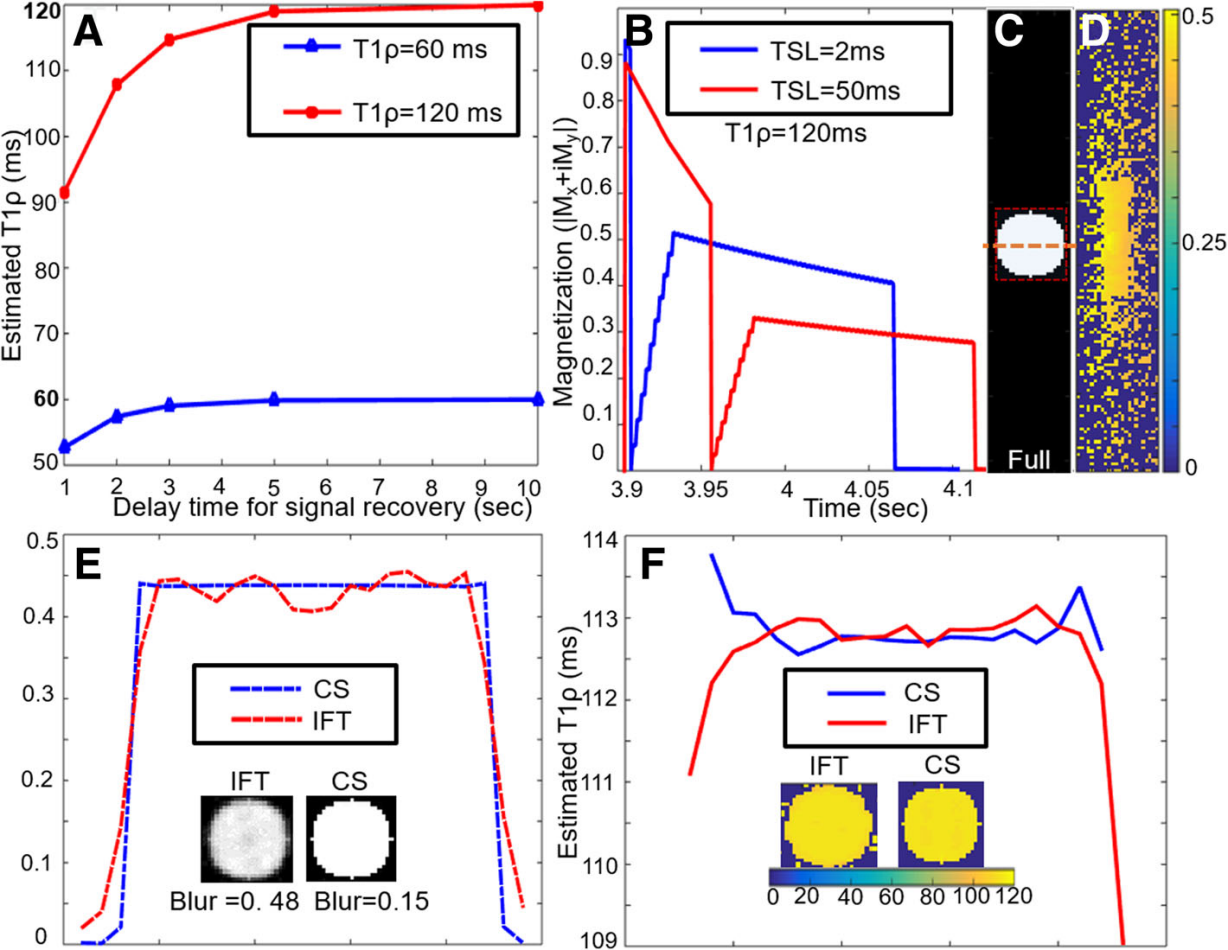
Results from Bloch simulation of the accelerated 3D T1ρ acquisition. **a** Curve showing the dependence of T1ρ relaxation error on heart rate. **b** The magnetization response curves showing the transverse magnetizations for TSL = 2 ms and 50 ms, **c** the fully sampled input image, **d** the k-space undersampling pattern modulated by the transient magnetization, **e** The plot of intensities across a horizontal line, (the location is shown using an orange dotted line in Fig. 2c) for the images reconstructed from the undersampled data using inverse Fourier transform (IFT) and using 3D TV constraints. The CS reconstruction has sharper edges as compared to IFT reconstruction. **f** The plot of estimated T1ρ values across the horizontal line for the T1ρ map estimated from the IFT and 3D CS reconstruction

The error in T1ρ due to incomplete recovery of magnetization between T1ρ preparations was 6.1% (normal) and 10.8% (infarct) at 60 bpm and 13.2% (normal) and19.6% (infarct) at 90 bpm. The dependence of T1ρ relaxation error on heart rate is shown in Fig. 2a for normal and infarcted tissue. A delay of 3 s between T1ρ-preparations reduced the percentage error in normal tissue to less than 2% of its true value and less than 4.2% in infarct.

The Bloch-simulated magnetization response is shown in Fig. 2b. The fully sampled image is shown in Fig. 2c and an example of an undersampling pattern modulated by the readout-dependent magnetization transients is shown in Fig. 2d. The images reconstructed from the undersampled and magnetization transience modulated k-space data are shown in Fig. 2e. The image reconstructed from the undersampled data using the inverse Fourier transform (IFT) suffers from edge blurring (blur metric = 0.48) while the image reconstructed using 3D TV constraints has a sharper edge profile (blur metric = 0.15). This result is further visualized in Fig. 2e using the plot of a vertical line across the image, the location is shown using an orange dotted line in Fig. 2c. The T1ρ map estimated from the IFT and CS reconstruction are shown in Fig. 2f. As seen in the plot of a line across the estimated T1ρ maps in Fig. 2f, blurring of edges in IFT reconstructions lead an increased error in the estimated T1ρ values at the edges of the cylinder, akin to the partial volume effect. The mean T1ρ estimated from IFT was (112.9 ± 0.2) ms and from CS reconstruction was (113.1 ± 0.2) ms. For the simulation of the normal myocardium, the mean T1ρ estimated from IFT was (67.4 ± 0.1) ms and from CS reconstruction was (67.1 ± 0.1) ms. The combined error from incomplete magnetization recovery, readout-dependent magnetization transients and undersampled k-space acquisition was 12% for healthy myocardium and 5.8% for infarct at 60 bpm.

### Phantom experiments

**Fig. 3:**
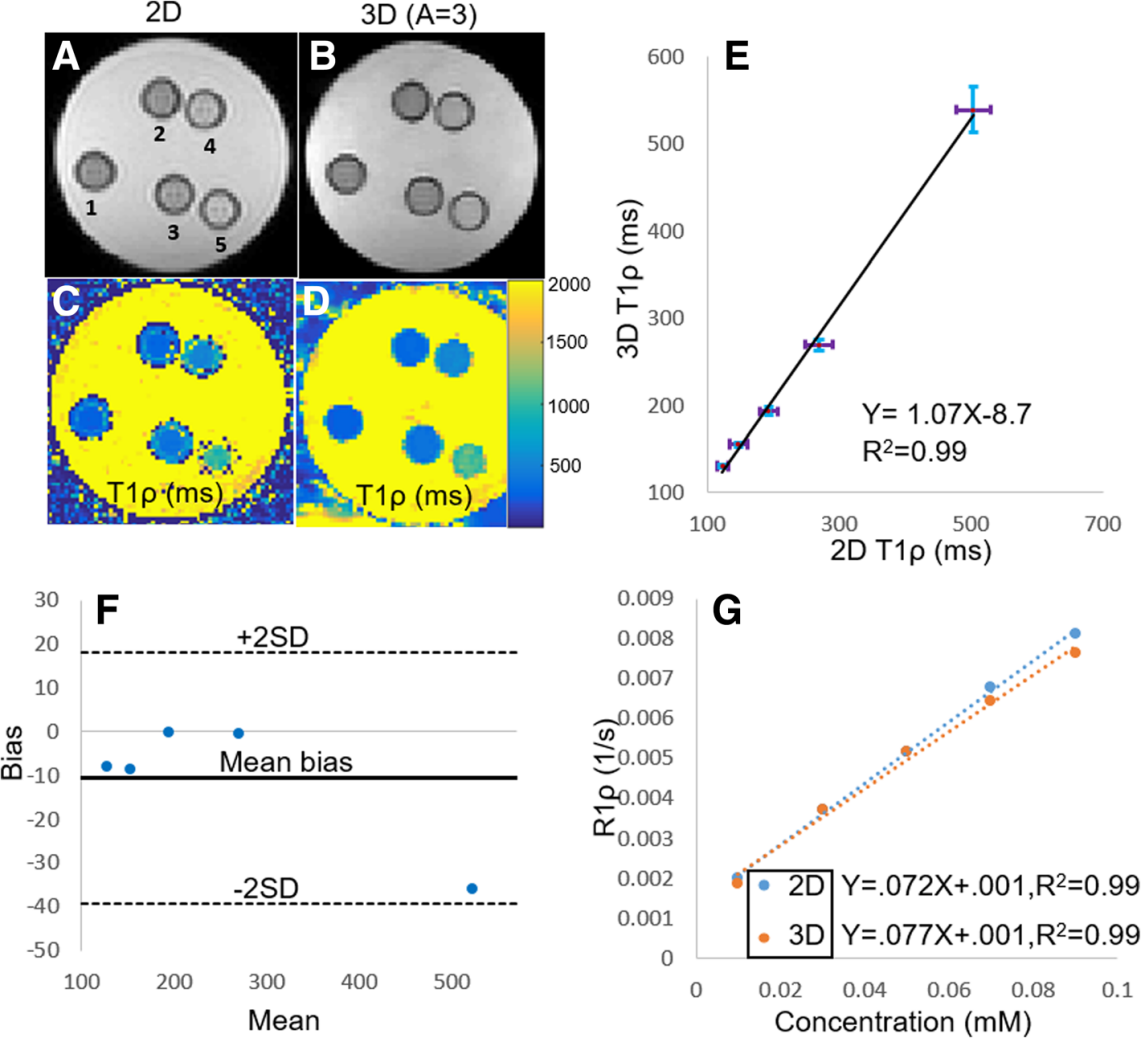
T1ρ-weighted images (TSL = 50 ms) (**a**, **b**) and maps (**c**, **d**) of saline phantoms doped with 1–5 wt% MnCl_2_. Images obtained using 2D single-shot (**a**, **c**) and accelerated (A = 3) compressed sensing (**b**, **d**) acquisitions. **e** Plot comparing the correlation between mean T1ρ estimated from the 2D and accelerated 3D images for the five MnCl_2_ doped phantoms. **f** Bland-Altman plot comparing the mean T1ρ (in ms) estimated from the 2D and accelerated 3D images. **g** Plot comparing correlation between R1ρ and concentration of MnCl_2_

To test the accuracy and repeatability of the accelerated 3D T1ρ scan, we prepared phantoms with different T1ρ relaxation times, scanned them three times, and reconstructed images and analyzed estimated T1ρ maps and compared to 2D T1ρ (Fig. 3a-d). There was excellent agreement between 2D and 3D T1ρ acquisitions (R^2^ = 0.99, *P* < 0.05; ICC = 0.99); Fig. 3e) and we were unable to detect a significant bias (Fig. 3f). R1ρ was highly correlated with MnCl_2_ concentration (*R*^2^ = 0.99; P < 0.05; Fig. 3g). The mean T1ρ from the three repeat acquisitions are reported in Additional file 2: Table S1. We did not detect a significant difference between the mean T1ρ estimated from the 3 repeat acquisitions (ICC = 0.99).

### Human experiments

**Fig. 4:**
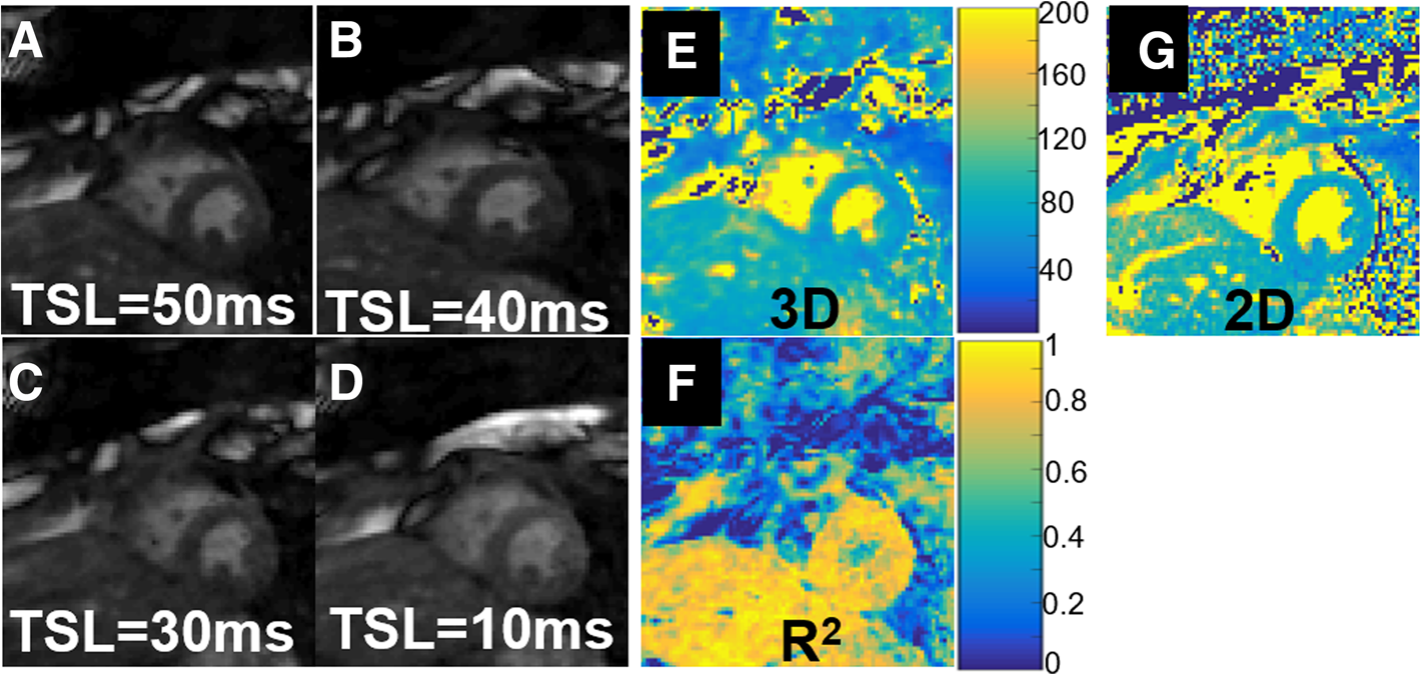
Results from an in-vivo human acquisition. T1ρ weighted images reconstructed using the proposed multicoil 3D total variation formulation is shown in (**a**-**d**). The myocardial T1ρ map (**e**) showed good uniformity and low least squares fit error (**f**). T1ρ map with the breath-held 2D sequence acquired at the same slice position is shown in (**g**). The mean T1ρ estimated from the free-breathing 3D scans and breath-held 2D were 67.9 ± 4.5 ms and 71.4 ± 6.5 ms, respectively

Example T1ρ images reconstructed using the multicoil 3D total variation formulation is shown in Fig. 4a-d. The convergence criterion was achieved in approximately 50 s, as shown in Additional file 3: Figure S2. A comparison of T1ρ weighted images reconstructed from the proposed undersampled 3D acquisition and a breath-held 2D acquisition is shown in Additional file 4: Figure S3. High quality T1ρ weighted images were reconstructed using the proposed multicoil 3D TV reconstruction formulation.

As expected in healthy subjects, the myocardial T1ρ map (Fig. 4e) showed good uniformity. A comparison with breath-held 2D T1ρ map, acquired at the same slice position, is shown in Fig. 4g. The T1ρ maps estimated from the free-breathing 3D scans and breath-held 2D scans showed good correspondence (3D T1ρ = (67.9 ± 4.5 ms) and 2D T1ρ = (71.4 ± 6.5 ms)). The mean T1ρ, COV and average navigator efficiency for the healthy subjects is reported in Additonal file 5: Table S2. The mean myocardial T1ρ estimated from the 2D and 3D scans matched well and there was no significant bias (mean bias = 0.87). The Bland-Altman plot based comparison of the estimated mean T1ρ is shown in Additional file 6: Figure S4. High quality images were reconstructed from the two repeat scans on healthy subjects. The comparison of the mean myocardial T1ρ showed that the proposed 3D technique is repeatable and the coefficient of variation of the myocardial T1ρ for 3D and 2D acquisitions on average were 7.1 and 6.4% respectively.

## Discussion

In this study, we developed an accelerated, free-breathing 3D T1ρ mapping technique that can provide full coverage of the left ventricle in human subjects. The significance of this work compared to existing CS 3D T1ρ mapping techniques [18–20, 22, 31] is that we have developed a T1ρ pulse sequence with variable-density undersampling on a clinical CMR scanner and prospectively assessed its accuracy and repeatability for cardiac imaging in phantoms and human subjects. Additionally, the reconstruction uses a novel combination of Split Bregman variable substitution and Fourier minimization for rapid reconstruction in a clinical setting.

A major contribution of this work is the analysis of the 3D T1ρ sequence using Bloch simulations to study the effect of incomplete magnetization recovery (heart rate), data undersampling and transient signal decay on edge blurring and relaxation time accuracy. Existing CS T1ρ techniques have been analyzed only in the setting of fully sampled k-space data that has been retrospectively undersampled and could not account for these factors. Since the k-space signal is dependent on magnetization transients, motion, and k-space ordering, it is necessary to prospectively acquire undersampled data to assess feasibility in human subjects. Existing methods assumed a uniform magnetization response and did not account for the transient effects of spatial encoding and k-space ordering on measured relaxation times.

The k-space ordering pattern used in this study tries to mitigate cardiac motion by acquiring k-space locations closest to the center of k-space in the same heart-beat/shot. Within a shot, a linear (column wise) sorting order was used to reduce the amount of eddy-current artifact due to rapid switching of gradients. In preliminary analysis, we found that by not splitting the low-frequency components of the k-space into multiple parts that were acquired over several heart beats, the motion artifacts could be better mitigated (data not shown). Since image quality can be affected by factors like eddy currents and motion, some novel k-space ordering techniques have been suggested [32–34]. In some studies [32, 33], the sampled k-space positions were first sorted based on the angular (radial) location of the points. The points within a shot were then sorted based on the radial distance from the center of k-space. This choice of k-space ordering was performed to minimize eddy current artifacts in applications such as contrast-enhanced whole-heart coronary CMR and accelerated 3D cine phase contrast imaging. In [34], the ill-effects of respiratory motion were sought to be mitigated by dynamically changing the location of acquired phase encode based on the location of the navigator.

In this study, a 3D TV constraint was chosen as the sparsifying transform for the CS implementation. This choice of constraint was to utilize the correlations in the data in the three spatial dimensions. The high performance of TV based constraints on undersampled cardiac data has been observed for several applications such as dynamic contrast enhanced cardiac perfusion imaging [35–38], cine imaging [39–41], and late gadolinium enhanced imaging of the left atrium [26, 42].

An acceleration factor of A = 3 was achieved by using a variable density sampling pattern in this study. Achieving high acceleration factors for free-breathing 3D mapping of the heart is a challenge due to the presence of cardiac and respiratory motion. In addition, Bloch simulation showed that variation in heart rate also has an effect on the estimated T1ρ.

Rapid multicoil CS minimization techniques have been proposed using methods such as FISTA [23], variable splitting [43], proximal operators [44], fast composite splitting algorithm (FCSA) [45, 46], SB/AL [17, 47], or a combination of techniques for applications such as cine imaging, dynamic contrast enhanced perfusion imaging and imaging of the brain. A detailed list of these techniques and their applications are shown in Additional file 7: Table S3. Depending on the application, these techniques have been developed to handle problems that arise due to a specific application of the type of constraint and nature of the data encoding matrix. The rapid reconstruction formulation developed here does not use FISTA-based iterative re-weighting for minimizing the quadratic L_2_ norm terms [37, 43, 44]. The Split Bregman- based variable substitution developed here uses one fewer variable substitution compared to [47] and does not need complex matrix factorization techniques used in [47, 48]. A detailed comparison of some of the existing rapid multicoil CS techniques is provided in [37].

CS 3D T1ρ techniques [18–20, 22, 31] have been developed for applications such as imaging of the knee cartilage, spine and brain. Since these techniques have been developed for different applications, each with their own set of challenges, there are differences in the sequence parameters such as acquired resolution, field-of-view, slice thickness, number of TSLs, and spatial encoding; in turn, these parameters affect the reconstruction performance. The parameter choices are customized to the needs of the application. A summary of these methods is discussed in Additional file 8: Table S4. In [18], a combination of principal component analysis and dictionary learning was used to reconstruct undersampled k-space data. The technique, called PANDA-T1ρ, was tested on retrospectively undersampled human brain and spine data with acceleration factors up to A = 4. A limitation of PANDA-T1ρ is the long reconstruction time and high computational complexity due to the need to train the dictionary at each iteration. In another study [19], a reconstruction technique developed for knee imaging used a sequential combination of a data-driven parallel imaging and the use of 2D spatial TV sparsifying transform. The CS reconstructions were performed on each coil data separately and minimized using a non-linear conjugate gradient solver. This technique was tested on two retrospectively undersampled in vivo human knee datasets for a net acceleration factor of 2.2, although slightly higher acceleration factors were reported on ex vivo porcine knee. The accelerated T1ρ mapping technique developed for imaging cartilage in [20] used an algorithm that alternated between local support detection in the space of principal components and joint image reconstruction and sensitivity estimation in SENSE (JSENSE). Feasibility of the technique was shown for acceleration factors up to 3.5 retrospectively in human knee cartilage. Accelerated simultaneous T2 and T1ρ MRI has been achieved using a blind compressed sensing (BCS) technique for brain mapping [31]. A limitation of this technique is the increased computational complexity and reconstruction time. A comparison of several CS reconstruction techniques with sparse and low rank models for T1ρ MRI has been recently reported [22]. The techniques were implemented using different variants of FISTA and combined with fast gradient projection (FGP) based algorithms. These were tested on phantom data and retrospectively undersampled in vivo knee cartilage data. The results showed that the implementations with spatiotemporal finite difference as sparsity constraint performed best for high acceleration factors.

Respiratory motion and cardiac motion are unique challenges in CMR that remain major factors in determining what acceleration factors may be achieved. Compared to applications such as imaging knee cartilage, human brain and spine, CMR parametric mapping has challenges in achieving very high acceleration rates. For example, for applications such as accelerated free-breathing 3D T1 mapping of the heart [49–51] the reported acceleration rates are comparable to what we have achieved. Existing accelerated 3D T1ρ techniques for non-CMR applications are analyzed by retrospectively undersampling fully sampled data. This approach is not suitable for in vivo cardiac applications as the fully sampled data is susceptible to intershot motion. Based on these factors, we chose to acquire prospectively undersampled data with an acceleration factor (A = 3), for which free-breathing 3D T1ρ mapping of the heart was feasible.

In order to estimate the feasibility of achieving higher acceleration factor for 3D cardiac T1ρ mapping, we performed experiments by retrospectively undersampling A= 3 data to *A* = 4 using a binary mask and compared the reconstructed images to *A* = 3. The results (in Additional file 9: Figure S5) show that in principle, it is feasible to achieve  A= 4, though with a marginal loss in image quality. Compared to  A= 3 images,  A= 4 images show additional smoothing of edges and loss of features. This shows that it will be difficult to achieve higher acceleration factors in clinical applications.

### Limitations

The study could be further improved by increasing the number of datasets. Unlike retrospectively undersampled techniques that can perform analysis at different acceleration factors (A = 2, 2.5 and 3), we did not acquire data at multiple acceleration factors due to the limited availability of patient scan time. Since the technique performs well at A = 3, the proposed technique should also work at lower acceleration factors. The effect of higher acceleration factors on free breathing 3D T1ρ would have to be further analyzed.

One major criterion that affected image quality was the constant change of the navigator position due to change in the patients breathing pattern or patient movement inside the scanner. One way to mitigate this problem would be to change the navigator acceptance window and position adaptively for each TSL acquired, though this could cause an increase in the total scan time.

Another limitation of the study was the long scan time needed to acquire the data. To estimate good quality T1ρ maps, images acquired using 12 TSL’s were used during curve fitting. This corresponded to a scan time of ~ 18 min at a 50% navigator efficiency. The scan time could be further reduced by increasing the navigator acceptance window width, though this could come at a cost of decrease in image quality of the reconstructed images due to motion.

### Future work

The reconstruction time of the technique can be accelerated by running on GPU’s or implementing on C++ instead of MATLAB. In addition, the reconstruction time could be further reduced by using coil compression techniques [52, 53] to reduce the total number of coils used for the reconstructions. The reconstructed image quality can be further improved by using additional constraints along the parameter dimension. The sampling pattern used to acquire the data from different TSL’s would have to be randomized so that artifacts do not overlap in the temporal dimension and sparsity can be effectively imposed. This can be applied in conjunction with motion compensation techniques to mitigate for cardiac motion between different TSL’s. This may also allow for further reduction in scan time by increasing the acceleration factor. One major drawback of simultaneously using constraints in the parameter dimension and spatial dimensions would be the increase in the reconstruction time, which would impede in the application of the reconstruction pipeline in a clinical framework. Though the technique has been developed for 3D T1ρ cardiac mapping, it is possible to extend the ideas from this work to other parametric mapping technique application with minimal modifications.

## Conclusions

This study shows that the proposed combination of multicoil 3D TV reconstruction and variable density based k-space undersampling allows for free-breathing T1ρ mapping of the whole heart. High quality T1ρ weighted images were rapidly reconstructed using the proposed SB based technique. The mean T1ρ estimated using the proposed technique matched well with the currently used 2D T1ρ mapping technique and there was no significant bias. This technique has the potential for clinical application of free-breathing T1ρ mapping.

## Additional files

Additional file 1: **Figure S1.** Pseudocode of the reconstruction algorithm. (DOCX 37 kb)

Additional file 2: **Table S1.** Results comparing the repeatability of T1ρ phantoms experiment. Mean, standard deviation (SD), coefficient of variation (COV) and results from the intra-class correlation coefficient (ICC) test is reported. (DOCX 12 kb)

Additional file 3: **Figure S2.** Plot showing the convergence of the cost functional. The relative change between successive iterations drops below 0.01% at the 48th iteration in ~ 51 s. (DOCX 735 kb)

Additional file 4: **Figure S3.** Figure comparing T1ρ weighted images from 2D ((A)-(F)) and 3D acquisitions ((G)-(L)). Good quality images were reconstructed from the undersampled 3D acquisitions. (DOCX 78 kb)

Additional file 5: **Table S2.** Results from in-vivo human datasets comparing 2D and 3D scans. Mean T1ρ (ms), COV and average navigator efficiency are reported. (DOCX 15 kb)

Additional file 6: **Figure S4.** Bland-Altman plot comparing the mean T1ρ (in ms) estimated from 2D and accelerated 3D images. (DOCX 42 kb)

Additional file 7:**Table S3.** An overview of some of the existing techniques that have been developed to rapidly minimize cost functionals of the form $C=\frac{\mu }{2}\sum \limits_{i=1}^{Nc}\parallel {EC}_im-{k}_i{\parallel}^2+\lambda {\left|{\phi}_1m\right|}_1+\eta {\left|{\phi}_2m\right|}_1$ is provided. (DOCX 54 kb)

Additional file 8: **Table S4.** An overview of the existing techniques proposed for accelerated CS T1ρ reconstructions. (DOCX 18 kb)

Additional file 9: **Figure S5.** Comparison of reconstructions from prospectively undersampled A = 3 data and A = 4 data that was created by retrospectively undersampling the *A* = 3 data using a binary mask (shown in (E)). The images reconstructed from A= 4 data ((B) and (D) have blurred edges, as compared to images reconstructed from A = 3 data (in (A) and (C)). (DOCX 202 kb)

## Abbreviations

ADMM: Alternating direction method of multipliers
BCS: Blind compressed sensing
bSSFP: Balanced steady-state free precession
CMR: Cardiovascular magnetic resonance
COV: Coefficient of variation
CS: Compressed sensing
ECG: Electrocardiogram
FCSA: Fast composite splitting algorithm
FGP: Fast gradient projection
FISTA: Fast iterative shrinkage-thresholding algorithm
GBCA: Gadolinium based contrast agent
ICC: Intra-class correlation coefficient
IFT: Inverse fourier transform
JSENSE: Joint image reconstruction and sensitivity estimation in sensitivity encoding
PANDA: Principle component analysis and dictionary learning
RF: Radiofrequency
SD: Standard deviation
SL: Spin lock
TSL: Spin lock time
TV: Total variation
