# Supplementary material for: Accelerated free-breathing 3D T1ρ cardiovascular magnetic resonance using multicoil compressed sensing
Source: J Cardiovasc Magn Reson. 2019 Jan 10;21:5. doi: 10.1186/s12968-018-0507-2 (PMC6327532; doi:10.1186/s12968-018-0507-2)
Supplement: Supplementary file 1 — Figure S1. Pseudocode of the reconstruction algorithm. (DOCX 37 kb) [file 12968_2018_507_MOESM1_ESM.docx]

To minimize

$${}_{\min P_{i},m,S,T,Q_{i}}\frac{\mu}{2}\sum_{i=1}^{Nc} \left\|EP_{i}-k_{i}\right\|_{2}^{2}+\lambda\left|S\right|_{1}+\frac{\alpha}{2}\sum_{i=1}^{Nc} \left\|P_{i}-C_{i}m-Q_{i}\right\|_{2}^{2}+\frac{\beta}{2}\left\|S-\nabla{}_{xyz}m-T\right\|_{2}^{2}$$

1. Initialize $j=0,m^{1}=\sum_{i}^{Nc} C_{i}^{*}E^{-1}d_{i},P_{i}^{1}=Q_{i}^{1}=S^{1}=T^{1}=0$

**Repeat**­

1. Compute$P_{i}^{(j+1)}$ from ${}_{\mathrm{argmin}P_{i}}\frac{\mu}{2}\sum_{i=1}^{Nc} \left\|EP_{i}-k_{i}\right\|_{2}^{2}+\frac{\alpha}{2}\left\|P_{i}-C_{i}m-Q_{i}\right\|_{2}^{2}$ using Fourier minimization.
2. Compute$m^{(j+1)}$ from ${}_{\mathrm{argmin}m}\frac{\alpha}{2}\sum_{i=1}^{Nc} \left\|P_{i}-C_{i}m-Q_{i}\right\|_{2}^{2}+\frac{\beta}{2}\left\|S-\nabla_{xyz}m-T\right\|_{2}^{2}$ using Fourier minimization.
3. Compute $S^{(j+1)}$ from ${}_{\min S}\lambda\left|S\right|_{1}+\frac{\beta}{2}\left\|S-\nabla{}_{xyz}m-T\right\|_{2}^{2}$using soft-thresholding/shrinkage.
4. Update$T^{(j+1)}=T^{(j)}-(\nabla{}_{xyz}m^{(j+1)}-S^{(j+1)})$.
5. Update $Q_{i}^{(j+1)}=Q_{i}^{(j)}-(C_{i}m^{(j+1)}-P_{i}^{(j+1)})$
6. Set j=j+1

**Until convergence**

Here *E* is the encoding matrix, *k_i_* is the measured undersampled k-space data, *m* is the estimated image, is the gradient operator in 3D and *C_i_* is the coil sensitivity map. *S* and *P_i_* are the surrogate variables and *Q_i_* and *T* come from optimizing the Bregman distance.
