# Supplementary material for: Accelerated free-breathing 3D T1ρ cardiovascular magnetic resonance using multicoil compressed sensing
Source: J Cardiovasc Magn Reson. 2019 Jan 10;21:5. doi: 10.1186/s12968-018-0507-2 (PMC6327532; doi:10.1186/s12968-018-0507-2)
Supplement: Supplementary file 2 — Table S1. Results comparing the repeatability of T1ρ phantoms experiment. Mean, standard deviation (SD), coefficient of variation (COV) and results from the intra-class correlation coefficient (ICC) test is reported. (DOCX 12 kb) [file 12968_2018_507_MOESM2_ESM.docx]

|  | **2D T1ρ** | | | | | **3D T1ρ (A=3)** | | | | |
| --- | --- | --- | --- | --- | --- | --- | --- | --- | --- | --- |
|  | Trial 1 | Trial 2 | Trial 3 | Mean ± SD (ms) | COV (%) | Trial 1 | Trial 2 | Trial 3 | Mean ± SD (ms) | COV (%) |
| 1 | 122.9±8.7 | 129.4±6.1 | 136.3±6.9 | 129.6±6.5 | 5.0 | 130.7±1.7 | 130.3±3.0 | 129.9±4.1 | 130.3±0.4 | 0.3 |
| 2 | 147.2±13.5 | 151.9±5.6 | 158.7±8.4 | 152.6±5.8 | 3.8 | 155.9±2.5 | 154.2±6.0 | 157.0±2.5 | 155.7 ±1.4 | 0.9 |
| 3 | 193.1±12.7 | 188.9±7.4 | 200.6±11.6 | 194.2±5.9 | 3.0 | 193.2±4.6 | 191.7±4.6 | 195.4±3.4 | 193.4±1.9 | 0.9 |
| 4 | 269.3±21.7 | 262.5±12.3 | 293.2±15.4 | 274.9±16.1 | 5.9 | 269.5±6.7 | 257.1±9.6 | 275.6±7.6 | 267.4±9.4 | 3.5 |
| 5 | 503.5±26.1 | 526.2±24.7 | 533.6±21.9 | 521.1±15.7 | 3.0 | 550.7±19.8 | 501.9±13.4 | 539.2±17.3 | 530.6±25.5 | 4.8 |
|  | ICC=0.99 | | | | | ICC=0.99 | | | | |
