# Supplementary figures and images for: Accelerated free-breathing 3D T1ρ cardiovascular magnetic resonance using multicoil compressed sensing
Source: J Cardiovasc Magn Reson. 2019 Jan 10;21:5. doi: 10.1186/s12968-018-0507-2 (PMC6327532; doi:10.1186/s12968-018-0507-2)

Iteration Number

Time=51 sec

**
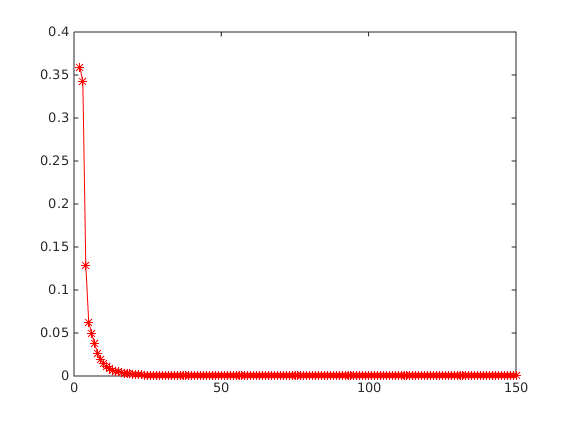
**

35

30

25

20

15

10

5

100

0

0

150

50

Supplement: Supplementary file 3 — Figure S2. Plot showing the convergence of the cost functional. The relative change between successive iterations drops below 0.01% at the 48th iteration in ~ 51 s. (DOCX 735 kb) [file 12968_2018_507_MOESM3_ESM.docx]

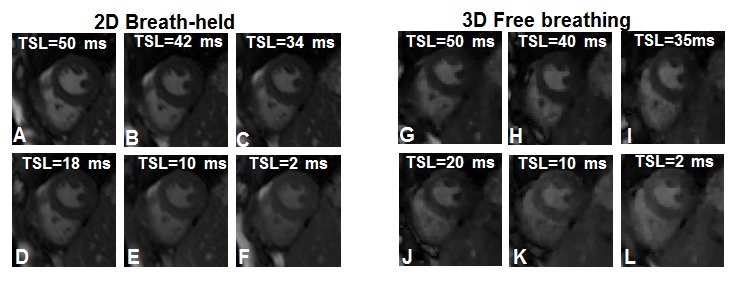

Supplement: Supplementary file 4 — Figure S3. Figure comparing T1ρ weighted images from 2D ((A)-(F)) and 3D acquisitions ((G)-(L)). Good quality images were reconstructed from the undersampled 3D acquisitions. (DOCX 78 kb) [file 12968_2018_507_MOESM4_ESM.docx]

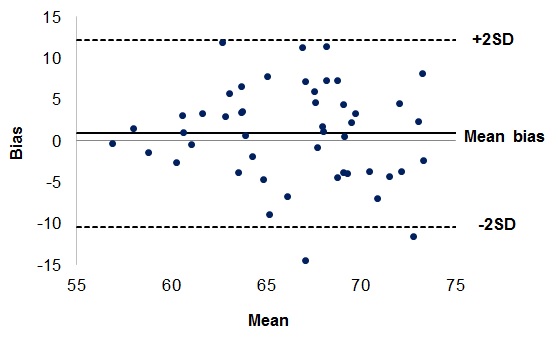

Supplement: Supplementary file 6 — Figure S4. Bland-Altman plot comparing the mean T1ρ (in ms) estimated from 2D and accelerated 3D images. (DOCX 42 kb) [file 12968_2018_507_MOESM6_ESM.docx]

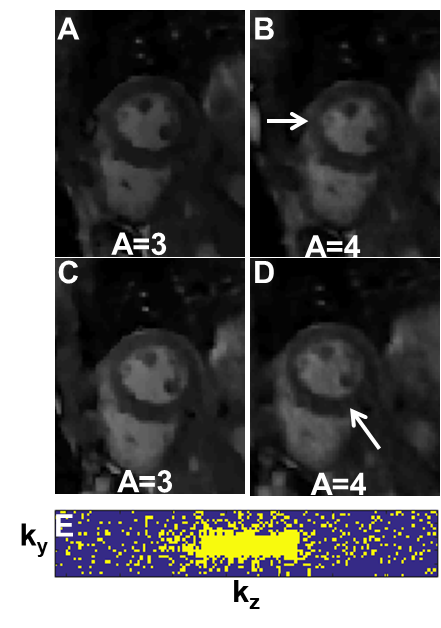

Supplement: Supplementary file 9 — Figure S5. Comparison of reconstructions from prospectively undersampled A = 3 data and A = 4 data that was created by retrospectively undersampling the A = 3 data using a binary mask (shown in (E)). The images reconstructed from A= 4 data ((B) and (D) have blurred edges, as compared to images reconstructed from A = 3 data (in (A) and (C)). (DOCX 202 kb) [file 12968_2018_507_MOESM9_ESM.docx]
