# Supplementary material for: Accelerated free-breathing 3D T1ρ cardiovascular magnetic resonance using multicoil compressed sensing
Source: J Cardiovasc Magn Reson. 2019 Jan 10;21:5. doi: 10.1186/s12968-018-0507-2 (PMC6327532; doi:10.1186/s12968-018-0507-2)
Supplement: Supplementary file 5 — Table S2. Results from in-vivo human datasets comparing 2D and 3D scans. Mean T1ρ (ms), COV and average navigator efficiency are reported. (DOCX 15 kb) [file 12968_2018_507_MOESM5_ESM.docx]

| Dataset | | R1 | R2 | R3 | R4 | R5 | R6 | Mean (ms) | COV (%) | Nav Efficiency (%) |
| --- | --- | --- | --- | --- | --- | --- | --- | --- | --- | --- |
| 1 | 2D | 74.2 | 77.3 | 69.4 | 68.6 | 64.2 | 72.5 | 71±4.6 | 6.5 | 71.7±9.2 |
|  | 3D | 71.9 | 69.2 | 68.9 | 72.3 | 63.6 | 61.3 | 67.9±4.5 | 6.6 |  |
| 2 | 2D | 71.3 | 74.3 | 68.8 | 70.5 | 68.6 | 70.6 | 70.7±2.1 | 2.9 | 52.9±12.4 |
|  | 3D | 68.1 | 69.8 | 66.8 | 64.6 | 67.5 | 63.6 | 66.7±2.3 | 3.5 |  |
| 3 | 2D | 70.3 | 71.3 | 72.4 | 61.1 | 63.3 | 67.4 | 67.1±5.4 | 8.1 | 41.6±6.7 |
|  | 3D | 74.4 | 66.9 | 65.1 | 61.2 | 65.3 | 74.4 | 67.8±5.2 | 7.8 |  |
| 4 | 2D | 74.1 | 73.9 | 70.6 | 67 | 59.8 | 63.3 | 67.7±5.4 | 8 | 40.1±4.1 |
|  | 3D | 64.6 | 62.5 | 68.4 | 78.6 | 74.3 | 60 | 67.9±7.3 | 10.7 |  |
| 5 | 2D | 72.1 | 69.3 | 65.6 | 61.6 | 59.6 | 63.8 | 65.5±4.2 | 6.3 | 49.5±5.6 |
|  | 3D | 74.5 | 73.7 | 62 | 65.5 | 59.1 | 61.4 | 66±6.5 | 9.9 |  |
| 6 | 2D | 65.9 | 58.6 | 69.9 | 68.9 | 64.9 | 67.3 | 66±3.9 | 5.9 | 63.7±13.1 |
|  | 3D | 60.1 | 61.6 | 65.3 | 61.2 | 62 | 68.1 | 63.1±3 | 4.7 |  |
| 7 | 2D | 62.9 | 61.7 | 63.2 | 61.1 | 66.4 | 69.5 | 64.2±3.56 | 5.8 | 48.3±5.5 |
|  | 3D | 69.6 | 67.2 | 57.3 | 59.5 | 57.1 | 71 | 63.6±6.4 | 10 |  |
| 8 | 2D | 67.1 | 66.9 | 60.7 | 68.6 | 62.8 | 67.3 | 65.6±3.1 | 4.7 | 68.2±7.5 |
|  | 3D | 71 | 60.4 | 61.3 | 56.8 | 69.5 | 71.3 | 65.1±6.3 | 9.6 |  |
