# Supplementary material for: Accelerated free-breathing 3D T1ρ cardiovascular magnetic resonance using multicoil compressed sensing
Source: J Cardiovasc Magn Reson. 2019 Jan 10;21:5. doi: 10.1186/s12968-018-0507-2 (PMC6327532; doi:10.1186/s12968-018-0507-2)
Supplement: Supplementary file 7 — Table S3. An overview of some of the existing techniques that have been developed to rapidly minimize cost functionals of the form $C=\frac{\mu }{2}\sum \limits_{i=1}^{Nc}\parallel {EC}_im-{k}_i{\parallel}^2+\lambda {\left|{\phi}_1m\right|}_1+\eta {\left|{\phi}_2m\right|}_1$C=μ2∑i=1Nc∥ECim−ki∥2+λϕ1m1+ηϕ2m1 is provided. (DOCX 54 kb) [file 12968_2018_507_MOESM7_ESM.docx]

|  | **Application** | **Type of constraint** | **Minimization technique developed** |
| --- | --- | --- | --- |
| Bilen et al. [1] | Cardiac perfusion imaging (retrospectively undersampled) | 1D Temporal TV and temporal wavelets | The cost function is minimized using a modified version of FISTA |
| Ye et al. [2] | Brain imaging (Retrospectively undersampled) | 2D Spatial TV and spatial wavelets | Enforcing *x*=*m* using variable splitting, cost functional can be rewritten as ${}_{\mathrm{argmin}m}\frac{\alpha}{2}\left\Vert m-x\right\Vert_{2}^{2}+\frac{\mu}{2}\sum_{i=1}^{NC} \left\Vert EC_{i}m-k_{i}\right\Vert_{2}^{2}$ ${}_{\mathrm{argmin}x}\left\Vert\varphi_{1}x\right\Vert_{1}+\left\Vert\varphi_{2}x\right\Vert_{1}+\frac{\alpha}{2}\left\Vert x-m\right\Vert_{2}^{2}$     Variable splitting is used to reduce the cost functional to a combination of a quadratic least squares and L_1_ norm denoising/filtering.  The L_2_ norm least squares sub-problem is minimized using FISTA and L_1_ norm based sub-problem minimized using SB and soft-thresholding. |
| Montefusco et al. [3] | Cardiac imaging | Application of 3D gradients, implemented using a 18-neighbor structure. | Assuming $x={}_{\mathrm{argmin}m}\sum_{i=1}^{NC} \left\Vert EC_{i}m-k_{i}\right\Vert_{2}^{2}$, the update is given by $x^{n}=m^{n}-\alpha\sum_{i=1}^{NC} (EC_{i})^{*T}(EC_{i}m-k_{i})$  Using proximal operators  $m^{n+1}={}_{\mathrm{argmin}m}\left\Vert\varphi m\right\Vert_{1}+\frac{\mu}{2\alpha}\left\Vert m-x^{n}\right\Vert_{2}^{2}$.  The reconstructions are accelerated using FISTA based iterative re-weighting. |
| Jiang et al. [4] | Brain imaging | 2D Spatial TV and spatial wavelets | The use of composite splitting to split the two constraints into individual L_1_ norm minimizations. ${}_{\mathrm{argmin}m}\left\Vert\varphi_{1}m\right\Vert_{1}+\frac{\mu}{2}\sum_{i=1}^{NC} \left\Vert EC_{i}m-k_{i}\right\Vert_{2}^{2}$ ${}_{\mathrm{argmin}m}\left\Vert\varphi_{2}m\right\Vert_{1}+\frac{\mu}{2}\sum_{i=1}^{NC} \left\Vert EC_{i}m-k_{i}\right\Vert_{2}^{2}$    Each sub-problem is minimized using FISTA. The final solution computed using a linear combination of the solution of the individual sub-problems. |
| Kamesh Iyer et al. [5] | DCE cardiac perfusion imaging (prospectively undersampled radial ) | 2D Spatial TV and 1D temporal TV | The following substitutions are enforced using SB: $P_{i}=C_{i}m$,$v_{1}=\varphi_{1}m$and $v_{2}=\varphi_{2}m$. ${}_{\mathrm{argmin}m}\lambda_{1}\left\Vert v_{1}\right\Vert_{1}+\lambda_{2}\left\Vert v_{2}\right\Vert_{1}+\frac{\mu}{2}\sum_{i=1}^{NC} \left\Vert EP_{i}-k_{i}\right\Vert_{2}^{2}+$     The L_1_ norm terms are minimized using soft-thresholding operator. The L_2_ norm terms are minimized using the iterative re-weighting used in FISTA. This technique was specifically designed for reconstructions that need to apply spatial and temporal TV constraints simultaneously. |
| Ramani et al. [6] | Brain imaging (retrospectively undersampled) | Spatial constraints | Enforcing *x=m*, *P_i_*=*C_i_m* and *v=ϕx* and enforcing using SB/AL        Closed form solutions are developed for computing$(\alpha_{2}I+\alpha_{3}\varphi^{T}\varphi)^{-1}$,$(\alpha_{2}I+\alpha_{1}C^{T}C)^{-1}$ and$(\alpha_{1}I+\mu E^{T}E)^{-1}$. The L_1_ norm terms are minimized using soft-thresholding. |
| Bilen et al. [7] | Cardiac imaging (retrospectively undersampled) | Wavelets/ Fourier transform /temporal TV | Enforcing *x*=*m* and *v= ϕx* using SB      Singular value decomposition (SVD) based technique is used to compute$(\alpha_{2}I+\mu(EC)^{T}EC)^{-1}$. Closed for solutions are available for $(\alpha_{2}I+\alpha_{1}\varphi^{T}\varphi)^{-1}$when wavelets or Fourier transform is used as sparsifying constraint while conjugate gradient (CG) based minimization is used when temporal TV is used as sparsifying transform. The L_1_ norm term is minimized using soft-thresholding |

**References**:

1. Bilen C, Selesnick IW, Wang Y, Otazo R, Kim D, Axel L, Sodickson DK: **On compressed sensing in parallel MRI of cardiac perfusion using temporal wavelet and TV regularization.** In *2010 IEEE International Conference on Acoustics, Speech and Signal Processing*; *14-19 March 2010*. 2010: 630-633.

2. Ye X, Chen Y, Lin W, Huang F: **Fast MR Image Reconstruction for Partially Parallel Imaging With Arbitrary k-Space Trajectories.** *IEEE Transactions on Medical Imaging* 2011, **30:**575-585.

3. Montefusco LB, Lazzaro D, Papi S, Guerrini C: **A Fast Compressed Sensing Approach to 3D MR Image Reconstruction.** *IEEE Transactions on Medical Imaging* 2011, **30:**1064-1075.

4. Jiang M, Jin J, Liu F, Yu Y, Xia L, Wang Y, Crozier S: **Sparsity-constrained SENSE reconstruction: An efficient implementation using a fast composite splitting algorithm.** *Magnetic Resonance Imaging* 2013, **31:**1218-1227.

5. Iyer SK, Tasdizen T, Likhite D, DiBella E: **Split Bregman multicoil accelerated reconstruction technique: A new framework for rapid reconstruction of cardiac perfusion MRI.** *Medical Physics* 2016, **43:**1969-1981.

6. Ramani S, Fessler JA: **Parallel MR Image Reconstruction Using Augmented Lagrangian Methods.** *IEEE Transactions on Medical Imaging* 2011, **30:**694-706.

7. Bilen C, Wang Y, Selesnick IW: **High-Speed Compressed Sensing Reconstruction in Dynamic Parallel MRI Using Augmented Lagrangian and Parallel Processing.** *IEEE Journal on Emerging and Selected Topics in Circuits and Systems* 2012, **2:**370-379.
