# Supplementary material for: Accelerated free-breathing 3D T1ρ cardiovascular magnetic resonance using multicoil compressed sensing
Source: J Cardiovasc Magn Reson. 2019 Jan 10;21:5. doi: 10.1186/s12968-018-0507-2 (PMC6327532; doi:10.1186/s12968-018-0507-2)
Supplement: Supplementary file 8 — Table S4. An overview of the existing techniques proposed for accelerated CS T1ρ reconstructions. (DOCX 18 kb) [file 12968_2018_507_MOESM8_ESM.docx]

|  | **Application** | **Reconstruction formulation** | **Acquisition parameters** | **In-vivo datasets** |
| --- | --- | --- | --- | --- |
| Pandit et al. [1] | Knee cartilage (retrospectively undersampled A=2.2). | A sequential combination of data driven parallel imaging and 2D spatial TV, applied on each coil independently. The cost functional was minimized using non-linear conjugate gradient (NL-CG) implementation. | Field strength=3T  Resolution=0.5mmX1mm  Matrix size=256X128  Slice thickness=4mm  Number of TSL’s=8  Spin lock freq=500 Hz  Readout= 3D spoiled gradient echo (SPGR) | 2 |
| Zhou et al. [2] | Knee cartilage (retrospectively undersampled A=3.5). | A combination of locally adaptive iterative support detection (LAISD) and estimation of coil sensitivities using JSENSE. Minimization was performed using an iterative weighted norm minimization technique. | Field strength=3T  Resolution=0.73mmX1.1mm  Matrix size=192X128  Slice thickness=4 mm  Number of TSL’s=8  Spin lock freq=500 Hz  Readout=Magnetization prepared angle modulated portioned kspace spoiled gradient echo snapshot (MAPSS) | 6 ( bilateral scans from 3 volunteers) |
| Zhu et al. [3] | Spine and brain imaging (retrospectively undersampled A=4). | A Combination of PCA and dictionary learning. | Spine imaging:  Field strength=3T  Resolution=1mmX1mm  Slice thickness=5 mm  Number of TSL’s=5  Spin lock freq=500 Hz  Readout=Turbo Spin Echo sequence (TSE)  Brain imaging:  Resolution=1mmX1mm  Slice thickness=5 mm  Number of TSL’s=5  Spin lock freq=500 Hz  b-SSFP sequence | 5 |
| Bhave et al. [4] | Single slice 2D Brain imaging (retrospectively undersampled A=10)  3D Brain imaging (prospectively undersampled A=8). | A Blind compressed sensing based technique for reconstructing images from undersampled 2D k-space data. A combination of variable splitting and augmented Lagrangian is used to accelerate convergence of the cost functional. The quadratic sub-problems are minimized using conjugate gradient and L_1_ norm term is minimized using soft thresholding. | Single slice 2D Brain imaging  Field strength=3T  Matrix size=128X128  Resolution=1.7mmX1.7mm  Slice thickness=5 mm  Number of TSL’s=12  Spin lock freq=330 Hz  Readout= TSE  3D Brain imaging  Matrix size=128X128  Resolution=1.7mmX1.7mmX1.7mm  Number of TSL’s=10  Spin lock freq=330 Hz  Readout= 3D MAPSS | 1  1 |
| Zibetti et al. [5] | Knee cartilage (retrospectively undersampled A=10). | Several sparse and low rank models were compared. The reconstructions were implemented using variants of FISTA. | Field strength=3T  Resolution=0.5mmX1mm  Matrix size=256X128X64  Slice thickness=2mm  Number of TSL’s=10  Spin lock freq=500 Hz  Readout= Turbo flash | 7 |

**References:**

1. Pandit P, Rivoire J, King K, Li X: **Accelerated T1ρ acquisition for knee cartilage quantification using compressed sensing and data‐driven parallel imaging: A feasibility study.** *Magnetic Resonance in Medicine* 2016, **75:**1256-1261.

2. Zhou Y, Pandit P, Pedoia V, Rivoire J, Wang Y, Liang D, Li X, Ying L: **Accelerating t1ρ cartilage imaging using compressed sensing with iterative locally adapted support detection and JSENSE.** *Magnetic Resonance in Medicine* 2016, **75:**1617-1629.

3. Zhu Y, Zhang Q, Liu Q, Wang YXJ, Liu X, Zheng H, Liang D, Yuan J: **PANDA‐ T1ρ: Integrating principal component analysis and dictionary learning for fast T1ρ mapping.** *Magnetic Resonance in Medicine* 2015, **73:**263-272.

4. Bhave S, Lingala SG, Johnson CP, Magnotta VA, Jacob M: **Accelerated Whole-Brain Multi-Parameter Mapping using Blind Compressed Sensing.** *Magnetic resonance in medicine* 2016, **75:**1175-1186.

5. Zibetti MVW, Sharafi A, Otazo R, Regatte RR: **Accelerating 3D‐T1ρ mapping of cartilage using compressed sensing with different sparse and low rank models.** *Magnetic Resonance in Medicine* 2018, **0:**1-18.
